# Believing emotions are uncontrollable is linked to eating disorder psychopathology via suppression and reappraisal

**DOI:** 10.1186/s40337-021-00395-8

**Published:** 2021-04-01

**Authors:** Laura Vuillier, Jemma Joseph, Matthew P. Somerville, Amy Harrison

**Affiliations:** 1 Faculty of Science and Technology, Department of Psychology, Bournemouth University, Poole, UK; 2 UCL Institute of Education, University College London, London, UK

**Keywords:** Emotional controllability, Emotion regulation, Reappraisal, Suppression, Eating psychopathology

## Abstract

**Objective:**

Research suggests that beliefs about emotional controllability influence the use of emotion regulation strategies, which in turn impact psychological health and illness. However, no research has yet investigated whether emotional controllability is linked to eating psychopathology. The current study investigates whether these concepts are related, as individuals with eating disorders have problems with emotion regulation.

**Method:**

We collected self-report data from 718 participants from a community sample using validated questionnaires, and ran mediational analyses to assess the relationship between emotional controllability and eating psychopathology, via reappraisal and suppression, two emotion regulation strategies.

**Results:**

Our mediational analyses suggest that believing emotions to be uncontrollable relates to high levels of suppression (*β* = −.08), low levels of reappraisal (*β* = .19) and poorer eating disorder psychopathology (*β* = −.11). Reappraisal and suppression were found to partially mediate the relationship between emotional controllability and eating psychopathology.

**Discussion:**

The current study has demonstrated relationships that support investigations relating emotional controllability, emotion regulation and psychological health. This research has potential implications for developing interventions to target beliefs about emotions in order to help improve emotion regulation skills and eating psychopathology.

**Supplementary Information:**

The online version contains supplementary material available at 10.1186/s40337-021-00395-8.

## Plain English summary

Everyone holds beliefs about the world and themselves. One of these beliefs concerns the ability to regulate emotions. Some people believe that they can control their emotions, while others believe that emotions are not changeable. Research into emotional controllability has found that believing emotions to be controllable is linked to better abilities to regulate them, and higher wellbeing. We also know from other research that people with eating disorders have difficulties regulating their emotions. However no research has looked at whether people with eating disorder behaviours view their emotions as uncontrollable and whether this has an influence on their abilities to regulate them. With the aid of 718 participants we found that, indeed, believing emotions to be uncontrollable was linked to poorer eating disorder behaviours. We also found that this relationship was partially explained by poorer emotion regulation strategies use. Our findings are important as they suggest practical implications for improving eating disorder psychopathology by changing beliefs about emotional controllability. However they are limited by the cross-sectional nature of the study, meaning we cannot imply causality. We recommend that further research replicate our findings in people with diagnosed eating disorders using a longitudinal design.

## Introduction

Emotional controllability refers to an individual’s beliefs about whether emotions are controllable or uncontrollable [1]. Recent studies suggest that beliefs about emotional controllability influence how people regulate their emotions, which in turn impacts psychological health [1, 2]. Emotional controllability is an area of emotional functioning that has been relatively under-researched in relation to eating disorders (EDs), yet we think these concepts could be related as individuals with EDs have problems with emotion regulation [3]. Identifying and exploring factors that relate to ED psychopathology is important due to the high comorbidity of EDs with other psychopathologies including mood, anxiety, personality, impulse-control, self-harm, and substance use disorders [4–6], and the high mortality associated with EDs [7]. This study seeks to contribute to this understanding by exploring relationships between emotional controllability and ED psychopathology, through emotion regulation.

### Emotional controllability

Believing that emotions are uncontrollable has been linked to experiencing more negative emotions, greater depression and lower levels of well-being. In a sample of male (*n* = 192) and female (*n* = 245) college students, Tamir et al. [1] found that, just before starting college, nearly 40% of the students believed emotions to be uncontrollable. After one academic year, individuals who held these beliefs had experienced more negative emotions and reported greater depression than those who had considered emotions to be controllable. These results were replicated by De Castella et al. [2] who revealed that increased stress and depression, and decreased self-esteem and satisfaction with life were reported by those who believed that emotions cannot be controlled.

### Emotion regulation and emotional controllability

Emotion regulation is a form of self-regulation that influences which emotions we experience, when they occur, how they are experienced, and how they are expressed [8]. While there are many strategies for regulating emotions, cognitive reappraisal and expressive suppression have received the most attention (e.g., [9]). Cognitive reappraisal (changing the way one *thinks* about emotion eliciting events) has been hypothesised to protect against psychopathology and is generally considered an adaptive strategy, while suppression (changing the way one *behaviourally responds* to the emotion) is thought to be a maladaptive strategy that has been considered a risk factor for psychopathology [10].

Emotional controllability has been found to influence an individual’s use of emotion regulation strategies like reappraisal [11]. Studies have consistently shown that individuals are less likely to reappraise their emotions when they believe them to be uncontrollable [1, 2, 11–13]. Ford et al. [12] noted this relationship fits with investigations into self-regulation that have suggested that engagement in self-regulation requires motivation, and the belief that it is achievable. As such, an individual who believes emotions are uncontrollable may not think that modifying them is achievable, and may, therefore, lack the motivation to use adaptive strategies like reappraisal.

King et al. [13] conducted recent research into emotional controllability, well-being and reappraisal, using a sample of 355 Filipino college students (females = 259, males = 82). Believing emotions to be uncontrollable was negatively related to life satisfaction and positive emotions (positive indicators of well-being), and positively related to negative emotions, depression and anxiety (negative indicators of well-being). The effect of emotional controllability on well-being was found to be partially mediated by reappraisal. This suggests that emotional controllability influences an individual’s reappraisal use, which then impacts their wellbeing.

Research into the relationship between emotional controllability and suppression has generated mixed results. Tamir et al. [1] and Ford et al. [12] found no difference in the use of suppression in individuals who viewed emotions as controllable vs uncontrollable. This suggests that emotional controllability is associated with strategies like reappraisal that aim to alter emotional *experiences*, not strategies like suppression that aim to alter emotional *expression* [12]. Yet this position is inconsistent with one of Schroder et al.’s [11] studies which did find a relationship between emotional controllability and suppression. However, this relationship was not consistent across their two experiments, rendering the significance of the relationship unclear. Further exploration of emotional controllability and suppression would be beneficial in providing clarity for the kind of strategies emotional controllability is associated with.

### Emotions and eating disorders

Problems with emotion regulation have been suggested to play a role in the development and maintenance of ED psychopathology [14–16]. The cognitive interpersonal maintenance model [17] has identified important predisposing roles of cognitive and socio-emotional factors in EDs. This model, with accumulating evidence to support it [18], suggests that EDs such as anorexia nervosa are maintained by difficulties surrounding emotional functioning. Examples of problems with emotional functioning that have been seen in individuals with EDs include alexithymia (the inability to identify and describe one’s feelings) [19, 20], increased negative emotionality [21], and difficulties regulating emotions [3, 21]. Importantly, difficulties with emotion regulation have been shown to correlate with the severity of ED psychopathology [15, 21–23].

Findings from Svaldi et al. [22] and Danner et al. [24] revealed that the severity of ED psychopathology in women negatively correlates with reappraisal use, and positively correlates with suppression use. However, Dixon-Gordon et al. [25] found that individuals who report a low use of all strategies (including reappraisal and suppression) experience amplified bulimia symptoms, making the use of suppression in EDs unclear and worthy of further exploration.

### The present study

The aforementioned literature suggests that emotional controllability influences the way people regulate their emotions, which then has an impact upon psychological health [1, 2, 13]. While emotional controllability has not yet been explored in relation to ED psychopathology, difficulties regulating emotions are known to play an important role in eating disordered behaviours [20, 21]. This raises the question of whether emotional controllability is associated with EDs via the use of emotion regulation strategies.

The main aim of the current study is to examine the relationship between emotional controllability, ED psychopathology and two emotion regulation strategies, namely suppression and reappraisal. We hypothesised a significant negative relationship between beliefs about emotional controllability and ED psychopathology mediated by the use of reappraisal and suppression independently. We also predicted relationships between reappraisal and ED psychopathology, as well as between suppression and ED psychopathology. That is, we expected those who report a high use of reappraisal to display less severe ED psychopathology as the use of adaptive strategies has been thought to protect against psychopathology [10] and is associated with better interpersonal functioning and well-being [9]. In addition, drawing on theories which suggest maladaptive strategies are a risk factor for psychopathology [10] and have been found to negatively correlate with interpersonal functioning and well-being [9], we expected more severe ED psychopathology to be displayed by those who report a high use of suppression.

## Method

### Participants

Originally, 884 participants completed the survey. However, 166 participants were excluded from the data set due to several missing responses in one or more questionnaires. The final sample included 718 participants (*n* = 556 females, *n* = 156 males, *n* = 6 other) from a range of ethnic groups (*n* = 398 Whites; n = 15 Blacks; *n* = 204 Asians; *n* = 35 Mixed; *n* = 59 Other; *n* = 7 not provided), with a mean age of 23.01 (SD = 8.18, age range = 56). Of the sample, 260 participants were students recruited from Bournemouth University, and the other participants (*n* = 458) were recruited online through a link distributed through email and social media (Facebook and Reddit).

### Measures

The predictor variable (emotional controllability) was operationalised using scores from two scales, the Implicit Beliefs about Emotion scale [1] and the Personal Beliefs about Emotion scale [2]. The outcome variable (ED psychopathology) was operationalised using the total score from the Eating Disorder Examination Questionnaire (EDE-Q [26];). The mediating variables (reappraisal and suppression) were operationalised using the scores from the Emotion Regulation Questionnaire (ERQ [9];).

#### Implicit beliefs about emotion scale

To assess general beliefs about the nature of emotion, we used Tamir et al.’s [1] Implicit Beliefs about Emotion Scale (IBES). The scale has shown good internal consistency in previous studies (α = .75, [1]; α = .77, [2]) and also in the current sample (α = .74). This scale correlates with the general controllability component of the Emotion Beliefs Questionnaire (*r* = −.45) [27], suggesting good external validity.

#### Personal beliefs about emotion scale

To assess personal beliefs about the nature of emotion, De Castella et al.’s [2] Personal Beliefs about Emotion Scale– an adapted version of the Implicit Beliefs about Emotion Scale [1] was used. The scales differ in terms of item wording, such that items embodying general beliefs use indefinite and third-person pronouns (e.g. “Everyone can learn to control their emotions”), whereas, personal beliefs are embodied by the use of first-person pronouns (e.g. “I can learn to control my emotions”). This scale shows good internal consistency (α = .79) [2],which was also confirmed in the current sample (α = .80). This scale correlated highly with the IBES in our sample (*r* = .70) and the results remained consistent across both scales. We report the results from the general belief scale (IBES) in the manuscript and the ones from the personal belief scale in the supplementary material.

#### Eating disorder examination questionnaire

Participants completed the Eating Disorder Examination Questionnaire (EDE-Q [26];) to assess their ED psychopathology severity. The EDEQ contains 28 questions referring to the past 28 days, such that high scores indicate severe ED psychopathology. The EDE-Q total score has strong internal consistency (α = .90) [28], which was confirmed in our sample (α = .96). This measure has also been shown to have good external validity in a community sample, (*r* = .84 with the Eating Disorder Examination Interview) [29], suggesting good external validity. Only the EDE-Q total score was examined as this study’s main aim was to investigate emotional controllability in relation to general ED psychopathology. As per Table 1, our sample was within the community norms reported in Carey et al. [30].

**Table 1:** Descriptive statistics of the five scales used

	Mean	SD	Range (Min-Max)
General EC (IBES)	3.2	0.80	4 (1–5)
Personal EC	3.4	0.87	4 (1–5)
EDE-Q	2.09	1.60	6 (0–6)
*EDE-Q N clinical range*	*n* = 121		
Reappraisal	4.60	1.5	6 (1–7)
Suppression	3.92	1.39	6 (1–7)

#### Emotional regulation questionnaire

Participants completed the Emotion Regulation Questionnaire (ERQ [9];) to evaluate their use of reappraisal and suppression. The scale consists of 10 questions with responses rating the agreeability of statements scored on a 7-point scale (1 = strongly disagree, 7 = strongly agree). The ERQ has demonstrated good external validity [31] as well as good internal consistency (reappraisal, α = .79; suppression, α = .73) [9], which was also confirmed in our sample (reappraisal, α = .86; suppression, α = .77).

### Procedure

The questionnaires were presented through the online platform Qualtrics (Qualtrics, Provo, UT) and began with demographic information, followed by the ERQ, the Emotional Controllability questionnaire and the EDE-Q. Participants also answered other questionnaires not reported in the current paper. This study received ethical approval from the Research Ethics Panel at Bournemouth University and University College London.

### Data analysis

A mediational analysis was conducted with all variables represented by continuous data. The PROCESS regression macro was used (via SPSS, version 25) as it uses bootstrapping, which is a nonparametric method that does not rely upon normally distributed data. It involves resampling the data set (5000 times) and estimating the indirect effect in each sample [32]. Research supports the use of bootstrapping as it provides accurate confidence limits [33], has good Type I error control and high power levels [34]. The effect size was calculated by means of percent mediation (P_M_ = (a*b)/c), to determine the degree to which the mediating variable is responsible for the total effect (c). This standardised method is independent of sample size, and the absence of a better alternative makes this a commonly used measure despite its limitations [35]. Model 1 looked at the relationship between personal emotional controllability and eating psychology through reappraisal while Model 2 looked at this relationship through suppression. We also ran these models with general rather than personal emotional controllability; results can be found in the supplementary material (Figs. 3 and 4).

## Results

The range, mean and standard deviations of the scores for each variable are presented in Table 1.

Average scores, followed by standard deviation, range and min-max (in brackets) on the Implicit Beliefs about Emotions scale (General EC), Personal Beliefs about Emotions scale (Personal EC), Eating Disorder Examination Questionnaire total score (EDE-Q), Reappraisal, and Suppression. For the EDE-Q, we added a row indicating the number of participants scoring in the clinical range (i.e. scoring above 4; EDE-Q N in clinical range).

### Mediation analysis of model 1

As predicted, there was a significant negative relationship between general emotional controllability and the severity of ED psychopathology (path c: *β* = −.11, *t*(716) = − 3.09, *p* = .002).

**Fig. 1:**
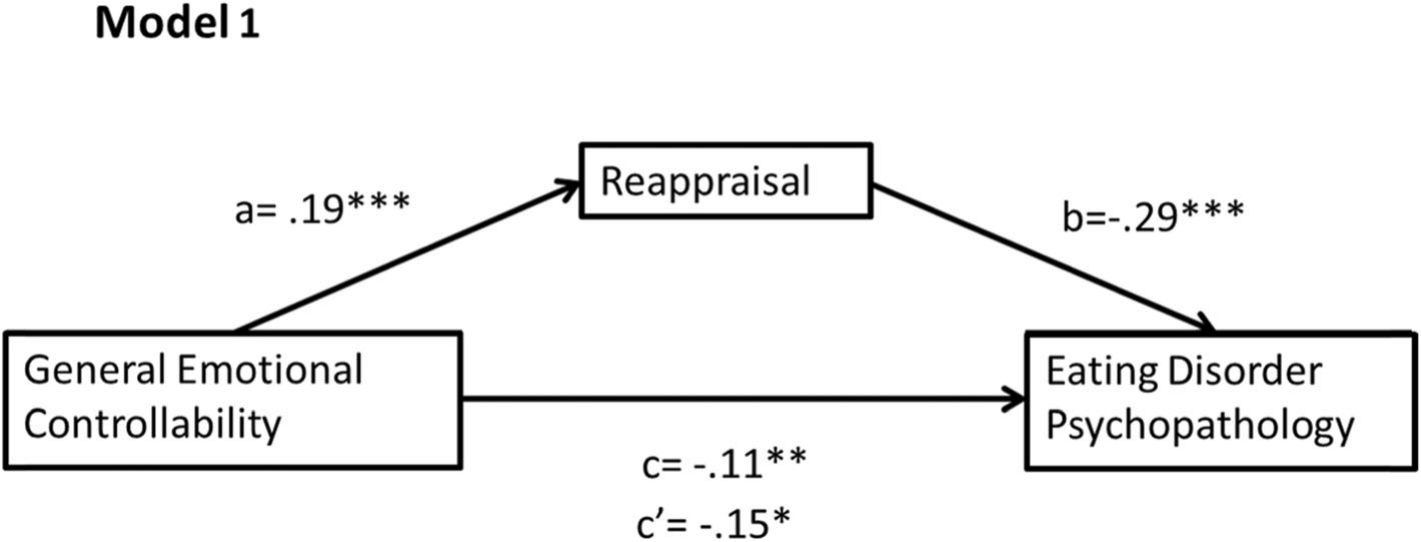
Mediation of Model 1, displaying standardised regression coefficients with their significance and the absolute value of c’ (* = *p* < .05, ** = *p* < .010, *** = *p* < .001)

The results for the mediation analysis of Model 1 are displayed in Fig. 1. The positive relationship between general emotional controllability and reappraisal was significant (path a: *β* = .19, *t*(716) = 5.10, *p* < .001), as was the negative relationship between reappraisal and ED psychopathology (path b: *β* = −.29, *t*(715) = − 5.63, *p* < .001). In addition, there was a significant indirect effect of emotional controllability on ED psychopathology through the use of reappraisal (*ab* = − 0.05, BCa CI [− 0.13, − 0.04]). When controlling for the mediating variable of reappraisal, the direct effect of emotional controllability on ED psychopathology was reduced but remained significant suggesting partial mediation (path c’: *β* = −.15, *t*(715) = − 2.04, *p* = .041). There was a moderate effect of reappraisal, *P*_*M*_ = .48, showing that reappraisal accounted for 48% of the effect of general emotional controllability on ED psychopathology.

### Mediation analysis of model 2

**Fig. 2:**
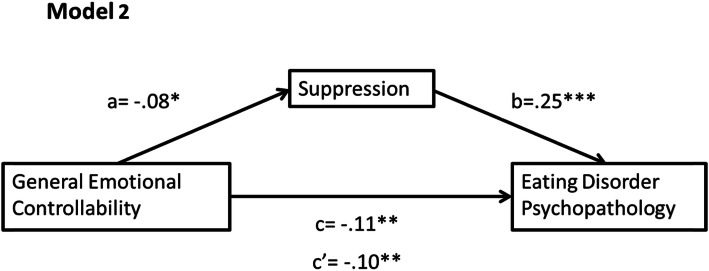
Mediation of Model 2, displaying standardised regression coefficients with their significance and the absolute value of c’ (* = *p* < .05, ** = *p* < .010, *** = *p* < *.*001)

The mediation analysis of Model 2 (Fig. 2) demonstrated a significant negative relationship between general emotional controllability and ED psychopathology through the mediation of suppression (*ab* = − 0.02, BCa CI [− 0.07, − 0.002]). There was a significant negative relationship between emotional controllability and suppression (path a: *β* = −.08, *t*(716) = − 2.12, *p* = .035), and a significant positive relationship between suppression and ED psychopathology (path b: *β* = .25, *t*(715) = 5.83, *p* < .001). Additionally, the direct effect of emotional controllability and ED psychopathology was reduced when controlling for suppression (path c’: *β* = −.10, *t*(715) = − 2.69, *p* = .007), but remained significant indicating partial mediation. There was a moderate effect of suppression, *P*_*M*_ = .17, showing that suppression accounted for 17% of the effect of general emotional controllability on ED psychopathology.

## Discussion

The aim of the present study was to explore the relationships between emotional controllability and ED psychopathology, and whether these relationships were mediated by emotion regulation. Through mediation analyses conducted on the self-report data from 718 participants, we found that (a) emotional controllability is linked to ED psychopathology, and (b) the relationship between emotional controllability and ED psychopathology is partially mediated by both reappraisal and suppression.

As predicted, the results demonstrated a significant negative relationship between emotional controllability and ED psychopathology severity. This supports the idea that viewing emotions as uncontrollable relates to poorer psychological health and well-being [1, 2, 12, 13], as individuals who held this view displayed greater severity of ED psychopathology. Moreover, the mediation analysis showed that this relationship was partially mediated by the use of reappraisal, partially confirming our hypothesis. Consistent with the findings from Tamir et al. [1], De Castella et al. [2], Schroder et al. [11], King et al. [13], and Ford et al. [12], results from the mediation analysis of Model 1 indicate a reduced use of reappraisal in individuals who perceived emotions as uncontrollable. The pattern of these findings suggests individuals who held the view that emotions are uncontrollable indicated a lack of reappraisal, and also more severe ED psychopathology. As discussed previously, an individual who believes emotions are uncontrollable may not think altering them was achievable, and in turn, may lack the motivation to use reappraisal. The insufficient use of adaptive strategies like reappraisal means an individual may not be able to effectively modify their emotional experiences, leading to emotion dysregulation [36]. This, in turn, may worsen the psychological and behavioural aspects of EDs, as previous research has demonstrated an association between emotion dysregulation and the severity of ED symptoms [21].

Interestingly, the results from the mediation analysis of Model 2 showed the use of suppression partially mediated the relationship between emotional controllability and ED psychopathology. A small negative relationship was found between emotional controllability and suppression, suggesting individuals who believe emotions to be uncontrollable display a *greater* use of suppression than those who believe emotions to be controllable. This challenges the findings that suggest emotional controllability only relates to strategies that target emotional *experience* [1, 12]. The current study also challenges the findings from Schroder et al. [11], which is the only known study to find an association between emotional controllability and suppression. Their findings suggest the belief that emotions are uncontrollable links to *little* use of suppression, whereas the findings in our study suggest this belief links to *high* use of suppression. It must again be noted that Schroder et al.’s [11] findings were inconsistent, with only their second study - conducted in a small and female only sample - finding a significant relationship between these concepts. Our findings suggest that an individual who views emotions as uncontrollable may not feel it is possible to change the negative emotions they experience, so may use suppression more frequently in order to alter the expression of emotions yet avoid having to engage with their emotional reactions.

### Limitations

A first limitation is that the findings are restricted to non-clinical samples, and may not be representative of the general population due to recruitment through opportunity sampling. As such, future studies could expand upon our findings by investigating whether our results replicate in a clinical sample. Ideally, this could be tested in a sample presenting a range of eating disorders (e.g. anorexia nervosa, bulimia nervosa and binge eating disorder) to explore potential differences between ED pathologies, and understand whether some eating disorder behaviours (e.g. dieting, bingeing or purging) may particularly correlate with emotional controllability. That being said, using non-clinical samples and investigating emotional functioning in ED psychopathology is beneficial. Firstly, in its own right, due to many individuals engaging in these behaviours [37], their higher prevalence compared to threshold eating disorders [38], and their links with psychopathology [39]. Secondly, some individuals exhibiting ED psychopathology may develop a full-threshold ED [38], meaning investigations with non-clinical samples could provide insight into the contribution of emotional functioning in the development of EDs [21].

Another limitation is the lack of generalisability arising from a predominately female sample, with a relatively young average age. As such, the findings may not be applicable to male dominated samples and older populations. In addition, the samples’ disproportionate female to male ratio may have impacted upon the findings given the higher prevalence of ED symptoms in females [40] and gender differences in emotion and emotion regulation. Generally, females are thought to be more emotional than males, expressing and experiencing them more regularly [41]. Moreover, while there are inconsistencies in the research surrounding gender differences in reappraisal and suppression use [12, 42–45], engagement in most forms of emotion regulation is more likely to be reported in females than males [46]. However, it is theorised that emotion regulation in males may be unconscious and automatic, and that their emotion regulation processes may as a consequence be inadequately informed in research [47], therefore suggesting fewer differences than originally thought. Future work may wish to investigate these gender differences, as well as explore a wider range of emotion regulation strategies such as avoidance, which is another strategy that has been related to both EDs [48] and emotional controllability [49].

Due to the cross-sectional nature of this study, interpretation of the results is also limited. Firstly because mediation tested in cross-sectional data may not always be replicated in longitudinal data [50, 51], so it is important to treat our current results with caution until such study is conducted. Second because our results cannot be interpreted in a way that suggests causation. The proposed models hypothesise the directions of the relationships between these variables, however, the directional relationships theorised may not be correct. For example, severe ED psychopathology may impact an individual’s ability to effectively regulate their emotions, which would guide their belief about their emotions. A longitudinal study would need to be conducted in order to confirm our findings and establish relationships of cause and effect. Nonetheless, the contributions of the current study should not be overlooked. This study has indeed challenged previous research into emotional controllability and suppression, supported existing research into emotion regulation strategies and ED psychopathology, and examined a link between emotional controllability and ED psychopathology that had not previously been investigated.

### Practical implications

The relationships and ideas suggested in this study have practical implications for improving ED psychopathology. Research has shown treatment addressing emotion regulation strategies decreases the rate of ED behaviours [52, 53]. In addition, Ford et al. [12] suggested targeting the belief that emotions are uncontrollable “may be a key ‘early’ step in the process of employing effective forms of emotion regulation” (p.1186). Blackwell et al.’s [54] findings suggest this approach can be successful as they demonstrated improvement in educational outcomes after interventions to improve adolescents’ beliefs about intelligence. Similar results have also been found in undergraduates [55]. Gutentag et al. [56] also found – in natural and unnatural settings – that the belief that emotions can be controlled relates to successful emotion regulation and more frequent use of effective emotion regulation strategies. From these findings, it could be reasoned that challenging the belief that emotions are uncontrollable may improve the use of effective emotion regulation strategies, which in turn may improve the psychological and behavioural aspects of EDs. Gutentag et al.’s [56] findings as well as ours will need replication, and further research is required, but support for this theory may have implications for ED treatments. If replicated and supported in a clinical sample, our results suggest that challenging emotional controllability beliefs may be an important component to add to the Emotional and Social Mind module of the Maudsley Anorexia Nervosa Treatment for Adults (MANTRA [49];) for example.

## Conclusion

The current study has demonstrated relationships that support investigating emotional controllability, emotion regulation and psychological health. Our findings suggest that one’s beliefs about emotional controllability has important outcomes with regards to eating psychopathology. While our results need to be replicated and tested in a clinical sample, our study lays the foundations for future research and possible treatment options in this area.

## Supplementary Information

**Additional file 1.**

## Data Availability

The datasets used and/or analysed during the current study are available from the corresponding author on reasonable request.

## Abbreviations

EC: Emotional Controllability
ED: Eating Disorder
EDE-Q: Eating Disorder Examination Questionnaire
ERQ: Emotion Regulation Questionnaire
