# Supplementary material for: Believing emotions are uncontrollable is linked to eating disorder psychopathology via suppression and reappraisal
Source: J Eat Disord. 2021 Apr 1;9:43. doi: 10.1186/s40337-021-00395-8 (PMC8015150; doi:10.1186/s40337-021-00395-8)

There was a significant negative relationship between personal emotional controllability and the severity of ED psychopathology (path c: *β*=-.23, *t*(716)=-6.19, *p*<.001). In the mediation analysis of Model 3 (Figure 3), the positive relationship between personal emotional controllability and reappraisal was significant (a: *β*=.32, *t*(716)=9.02, *p*<.001), as was the negative relationship between reappraisal and ED psychopathology (b: *β*=-.17, *t*(715)=-4.42, *p*<.001). In addition, there was a significant indirect effect of emotional controllability on ED psychopathology through the use of reappraisal (*ab*=-0.05, BCa CI [-.15, -.05]). When controlling for the mediating variable of reappraisal, the direct effect of emotional controllability on ED psychopathology was reduced but remained significant suggesting partial mediation (c’: *β*=-.17, *t*(715)=-4.53, *p*<.001). There was a moderate effect of reappraisal, *P_M_*=.24, showing that reappraisal accounted for 24% of the effect of personal emotional controllability on ED psychopathology.

Figure 3. Mediation of Model 3, displaying regression coefficients with their significance and the absolute value of c’ (***=*p*<.001).


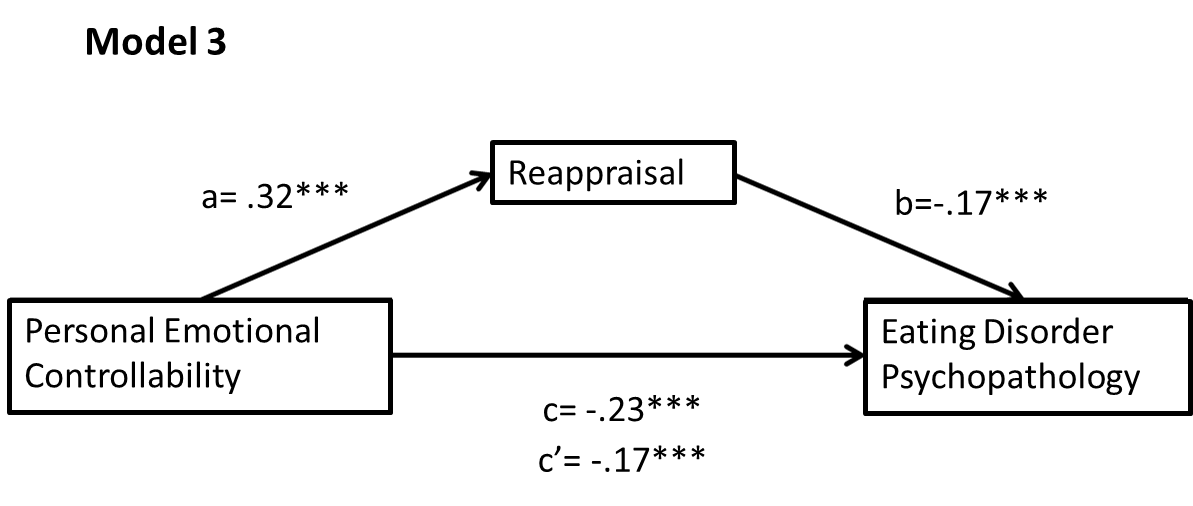


The mediation analysis of Model 4 (Figure 4) demonstrated a significant negative relationship between personal emotional controllability and ED psychopathology through the mediation of suppression (*ab*=-0.02, BCa CI [-.08, -.01]). There was a significant negative relationship between emotional controllability and suppression (path a: *β*=-.12, *t*(716)=-3.23, *p*=.0013), and a significant positive relationship between suppression and ED psychopathology (path b: *β*=.20, *t*(715)=5.44, *p*<.001). Additionally, the direct effect of emotional controllability and ED psychopathology was reduced when controlling for suppression (path c’: *β*=-.20, *t*(715)=-5.62, *p*<.001), but remained significant indicating partial mediation. There was a moderate effect of suppression, *P_M_*=.10, showing that suppression accounted for 10% of the effect of personal emotional controllability on ED psychopathology.

Figure 4. Mediation of Model 4, displaying regression coefficients with their significance and the absolute value of c’ (**=*p*<.010, ***=*p*<.001).


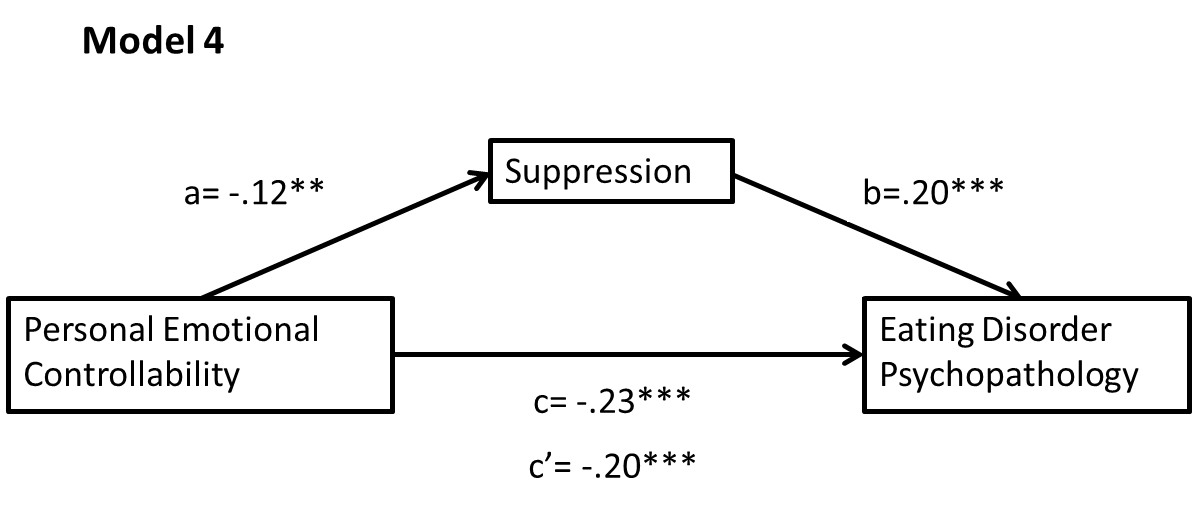

Supplement: Supplementary file 1 — Additional file 1. [file 40337_2021_395_MOESM1_ESM.docx]
